# Angiotensin-Converting Enzyme Genotype–Specific Immune Response Contributes to the Susceptibility of COVID-19: A Nested Case–Control Study

**DOI:** 10.3389/fphar.2021.759587

**Published:** 2022-01-12

**Authors:** Pengyun Gong, Fanghua Mei, Ruili Li, Yuchen Wang, Weizheng Li, Kai Pan, Junqiang Xu, Chao Liu, Hongjun Li, Kun Cai, Wei Shi

**Affiliations:** ^1^ School of Engineering Medicine and School of Biological Science and Medical Engineering, Beihang University, Beijing, China; ^2^ Hubei Center for Disease Control and Prevention, Wuhan, China; ^3^ Department of Radiology, Beijing YouAn Hospital, Capital Medical University, Beijing, China

**Keywords:** angiotensin-converting enzyme, immune response, genotype, COVID-19, cytokine

## Abstract

**Background:** Severe acute respiratory syndrome coronavirus 2 (SARS-CoV-2) is the cause of coronavirus disease 2019 (COVID-19), which has resulted in a global pandemic.

**Methodology:** We used a two-step polymerase chain reaction to detect the ACE genotype and ELISA kits to detect the cytokine factor. We also used proteomics to identify the immune pathway related to the ACE protein expression.

**Result:** In this study, we found that the angiotensin-converting enzyme (ACE) deletion polymorphism was associated with the susceptibility to COVID-19 in a risk-dependent manner among the Chinese population. D/D genotype distributions were higher in the COVID-19 disease group than in the control group (D/D odds ratio is 3.87 for mild (*p* value < 0.0001), 2.59 for moderate (*p* value = 0.0002), and 4.05 for severe symptoms (*p* value < 0.0001), logic regression analysis. Moreover, genotype-specific cytokine storms and immune responses were found enriched in patients with the ACE deletion polymorphism, suggesting the contribution to the susceptibility to COVID-19. Finally, we identified the immune pathway such as the complement system related to the ACE protein expression of patients by lung and plasma proteomics.

**Conclusion:** Our results demonstrated that it is very important to consider gene polymorphisms in the population to discover a host-based COVID-19 vaccine and drug design for preventive and precision medicine.

## Main

Severe acute respiratory syndrome coronavirus 2 (SARS-CoV-2) is the virus that causes coronavirus disease 2019 (COVID-19), which was first reported in December 2019 (Chan et al., 2020; Huang et al., 2020; Zhu et al., 2020). SARS-CoV-2 has infected over 103 million people in 216 countries/regions, causing over 2,222,506 deaths as of June 31st, 2021 (https://coronavirus.jhu.edu/map.html). SARS-CoV-2 is a highly contagious positive-strand RNA virus, and its clinical outputs include severe respiratory disease that appears to be fatal in 2–3% of patients.

In respiratory diseases, such as COVID-19, the host genetic factors thought to define resistance or susceptibility to infection are important. Several previous studies have reported that an ACE2 polymorphism, the direct receptor of SARS-CoV-2, may be involved in the susceptibility to the infection (Ghafouri-Fard et al., 2020). Here, we present ACE, a paralog of ACE2, which also plays an important role in the treatment of COVID-19. ACE2 and ACE co-regulate sodium and potassium balance and blood pressure in the renin–angiotensin–aldosterone system (RAAS). Specifically, ACE produces AngII, and ACE2 degrades AngII to Ang1–7. Some previous studies showed that ACE polymorphism, which was defined in detail by the absence or presence of a 287-bp DNA fragment in intron 16 of the ACE gene, was associated with various heart-related and other diseases such as atherosclerosis (Sayed-Tabatabaei et al., 2003), myocardial infarction (Cambien et al., 1992), ischemic stroke (Sharma, 1998), diabetic nephropathy (Boright et al., 2005), hypertension (Pachocka et al., 2020), endurance exercise (John et al., 2020), and cancer (Raba et al., 2020). Recently, ACE inhibitors have been used to treat hypertension with a lower proportion of critical patients and a lower death rate in COVID-19 patients (Yang et al., 2020). These results inspired the investigation of the association between ACE polymorphism and COVID-19.

The elegant RAAS also plays a significant role in the maintenance and promotion of inflammation (Pacurari et al., 2014), and the cytokine storm caused by the inflammation of SARS-CoV-2 is the major cause of death in COVID-19 patients (Huang et al., 2020). The imbalance of the ACE2/AngII/AT1R axis and their complementary cascades are the key molecular interactions that support the cytokine storm (Hirano and Murakami, 2020). Once ACE2 is occupied by the virus infection; this enzyme does not convert AngII into Ang1–7, inducing the difficulty to activate MasR to control the inflammation (Kuba et al., 2005; Mahmudpour et al., 2020). Since ACE is a core enzyme in RAAS that can produce AngII from AngI, ACE polymorphism was associated with inflammation hypothetically. However, the current data about the role of genetic risk factors, especially RAAS and ACE polymorphism, in the determination of the rate of SARS-CoV-2 infection in each ethnic group are limited and have not been validated by experimental assays.

To investigate whether the ACE polymorphism is involved in the development of COVID-19, 419 COVID patients and 441 control subjects from Hubei Province were studied. Meanwhile, ACE serum activities, the receptor-binding domain (RBD) of SARS-CoV-2 antibodies, and cytokines were tested based on the gene polymorphism of Chinese populations. Moreover, we performed proteomic research and presented genotype-specific immune responses and protective roles of the II genotype for the patients infected with COVID-19. Therefore, it is very important to consider the gene polymorphisms in the population to discover host-based vaccines and drug designs with personalized therapy.

## Result

### ACE DD Polymorphism Acting as a Potential Risk Factor for COVID-19

Mild, moderate, and severe patients diagnosed with COVID-19 according to the nucleic acid test and chest CT over the study period (January 2020 to March 2020) were eligible. Blood samples were taken 3–10 days after being diagnosed with COVID-19. Healthy control subjects without personal or family history of premature coronary and hypertension disease, as well as other cardiovascular-related diseases, were recruited for the cohort study (Supplementary Table S1). We investigated whether the insertion/deletion polymorphism in the ACE gene was associated with the susceptibility to COVID-19. We used the two-step polymerase chain reaction (PCR) to avoid the preference for the DD genotypes over ID genotypes due to the length difference. This protocol warrants that in every patient the correct genotype is determined (Biller et al., 2006). Table 1 shows the genotypic frequencies of the ACE insertion/deletion polymorphism. Genotypic frequencies of D/D, D/I, and I/I were 30.55, 41.77, and 27.68 vs. 12.93, 51.70, and 35.37% (COVID-19 patients vs. control subjects), respectively. Notably, the distribution of ACE polymorphism in the control group was similar to previous meta-analysis studies in the Chinese population (Huang et al., 2016). D/D genotype distributions were higher in the COVID-19 disease group than in the control group [D/D odds ratio is 3.87 for mild (*p* value < 0.0001), 2.59 for moderate (*p* value = 0.0002), and 4.05 for severe symptoms (*p* value < 0.0001), logic regression analysis, Table 1], showing that the ACE insertion/deletion polymorphism was associated with COVID-19 in the Chinese population.

**TABLE 1:** Distribution of the ACE genotype in different symptoms of COVID-19 patients.

ACE genotype	Mild	Moderate	Severe	Healthy
DD	36	56	36	57
ID	40	94	43	228
II	28	64	24	156
Odds ratio	3.57****	2.39****	3.73****	
	(2.20–5.84)	(1.59–3.57)	(2.29–6.15)	
D/I	0.55/0.45	0.48/0.52	0.56/0.44	0.37/0.63

Statistical analysis of the association between COVID-19 and ACE polymorphism (logistic regression analysis); ACE genotype (DD more frequent in patients than in controls) and symptom (different relative frequency of patients in three symptoms): ****p value < 0.0001, ***p value < 0.001.95% confidence intervals are given within brackets. The odds ratio is a measure of the relative risk of COVID-19 between patients with the DD genotype and patients with the ID or II genotypes.

As the influence of the DD genotype seemed to be independent of classical risk factors (Cambien et al., 1992), we considered that it was a potentially important factor for COVID-19 patients with low risk according to the usual criteria. Since a previous study reported that age and sex may be two independent usual criteria for susceptibility of SARS-CoV-2 infection (Bhatraju et al., 2020), a low-risk group was defined according to age (<50 years) and sex (female). In this group (Table 2), the association between ACE polymorphism and COVID-19 was significant. The odds ratio (an estimate of relative risk) was 6.40 (*p* value = 0.039) for severe symptoms of COVID-19 in the low-risk group. Meanwhile, in the high-risk group, which included male subjects aged >50 years, the odds ratio was 1.55 for severe symptoms of COVID-19 (*p* value = 0.60, multiple regression analysis, Table 2). These results indicated that the DD genotype was associated with COVID-19 in a risk-dependent manner.

**TABLE 2:** Distribution of the ACE genotype for different symptoms of COVID-19 patients according to risk status.

ACE genotype	Mild	Moderate	Severe	Healthy
Low risk (female, age<50)				
DD	11	22	4	5
ID + II	15	37	4	32
Odds ratio	4.69*	3.81*	6.40*	—
High-risk (male, age>50)				
DD	13	16	13	6
ID + II	21	39	42	30
Odds ratio	3.10	2.05	1.55	—

Statistical analysis of the association between COVID-19 and ACE polymorphism (multiple logistic regression analysis): ACE genotype (DD, more frequent in patients than in controls); symptom (different relative frequency of patients in the three symptoms); and risk group (high-risk status more frequent in cases than in controls). Heterogeneity of ACE and the genotype effect according to the risk group (DD genotype more strongly associated with COVID-19 in mild symptoms of the high-risk group and in severe symptoms of the low-risk group, *p value < 0.05).

**FIGURE 1:**
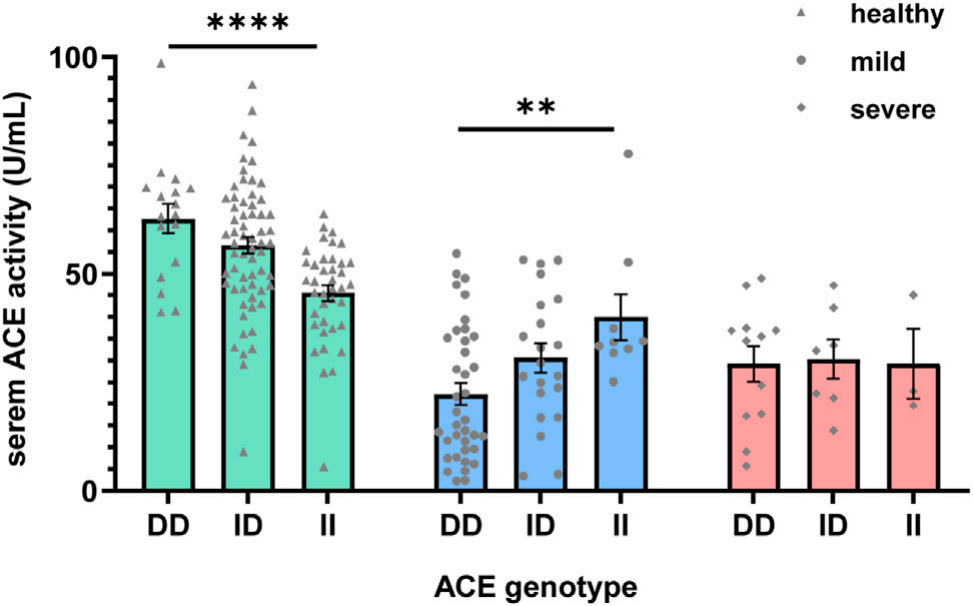
Mean serum angiotensin-converting enzyme (ACE) levels of COVID-19 patients with different ACE genotypes and different symptoms. Serum ACE levels and the ACE genotypes showed significant correlation with different symptoms of the COVID-19 patients [two-way ANOVA, F (4, 198) = 6.743, *p* value < 0.0001]. The mean serum ACE level without any regard to the genotype reduced during the COVID-19. Data are presented as mean SEM.

Next, we investigated the ACE serum activity of COVID-19 patients in a genotype-specific manner. We found reduced ACE serum activity in the DD and ID genotypes of the COVID-19 patients compared to that of the healthy subjects of the same genotyping (Figure 1). Similar to previous studies (Biller et al., 2006), we found that the DD genotype have the highest ACE serum activity in the control group [DD = 62.67 ± 3.13 U/ml, *n* = 17; ID = 56.54 ± 1.85 U/ml, *n* = 62; II = 45.53 ± 1.76 U/ml, *n* = 39, one-way ANOVA, F (2, 115) = 11.73, *p* value < 0.0001]. There was a significant difference between different genotype groups in the mild symptoms (ACE serum activity: DD = 22.23 ± 2.53 U/ml, *n* = 37; ID = 30.61 ± 3.32 U/ml, *n* = 21; II = 39.92 ± 5.31 U/ml, *n* = 9, one-way ANOVA, F (2, 64) = 5.495, *p* value = 0.006), with the highest ACE activity in the II genotype. There was also a significant difference between the disease and genotypes of the two factors [two-way ANOVA, F (4, 198) = 6.743, *p* value < 0.0001]. These results suggest that the genotype-specific differential expression of ACE activity may result in the different stages of lung injury.

### Genotype-Specific Cytokines and Immune Response After Infection of COVID-19

**FIGURE 2:**
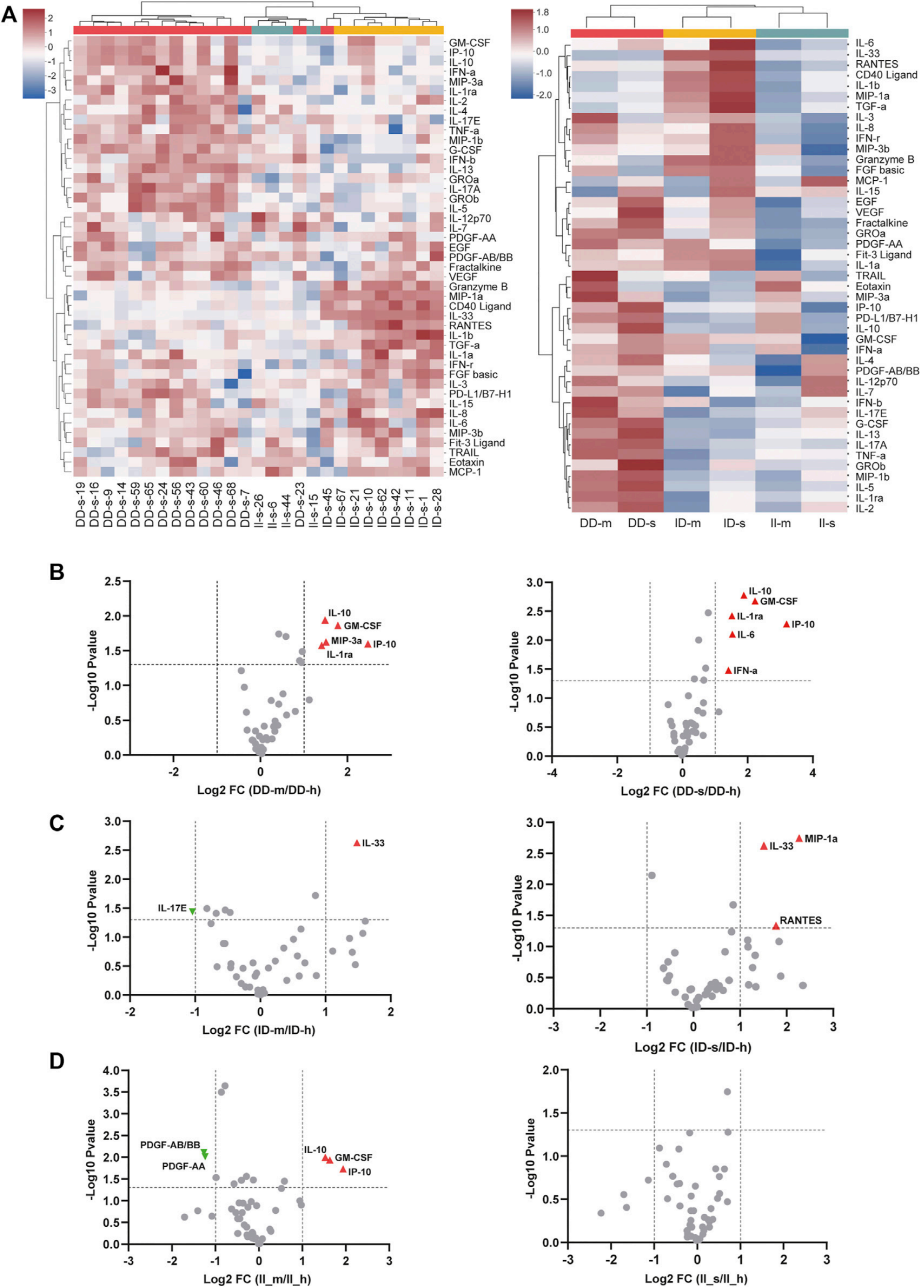
Cytokines and chemokines in COVID-19 patients with mild and severe symptoms in Hubei Province. **(A)** The patients were grouped according to ACE DD, ID, and II genotypes. Each genotype contained two subgroups based on different progressions of pneumonia. Healthy, mild, and severe patients are indicated as h, m, and s, respectively. We removed the outliers and replaced them with the median of other values in the same subgroups. The cytokines of the mild and severe subgroups were divided by the healthy subgroups. The calculated results were averaged and then we took the logarithm (log_2_) of the results in order to unify the dimension. **(B–D)** To obtain the aforementioned statistical results, we calculated the negative logarithm (log_10_) of the *p* value for each subgroup to indicate the significance of the differences between two groups of data (−log_10_ (*p* value) ≥ 1.30103 means significant). Volcano plots were shown to characterize the cytokine differences between the patients and the healthy groups in different ACE genotypes.

To further investigate the mechanism of the DD genotype in the susceptibility of COVID-19 disease, we tested cytokines and chemokines in COVID-19 patients with mild and severe symptoms. Similar to previous studies (Huang et al., 2020), we found that there were higher serum IL-1B, IL-2, IL-6, IL-8, IL-10, GMCSF, IFN, IP10, MIP1, TNF, and VEGF concentrations in both mild and severe patients than the healthy control group. Interestingly, we found that patients with the DD genotype had higher cytokine storms and chemokine responses than patients with II genotype in both mild and severe groups in Hubei Province (Figure 2A). In addition, we used a volcano plot to characterize the cytokine differences between the patients and the healthy groups (Figures 2B–D).

**FIGURE 3:**
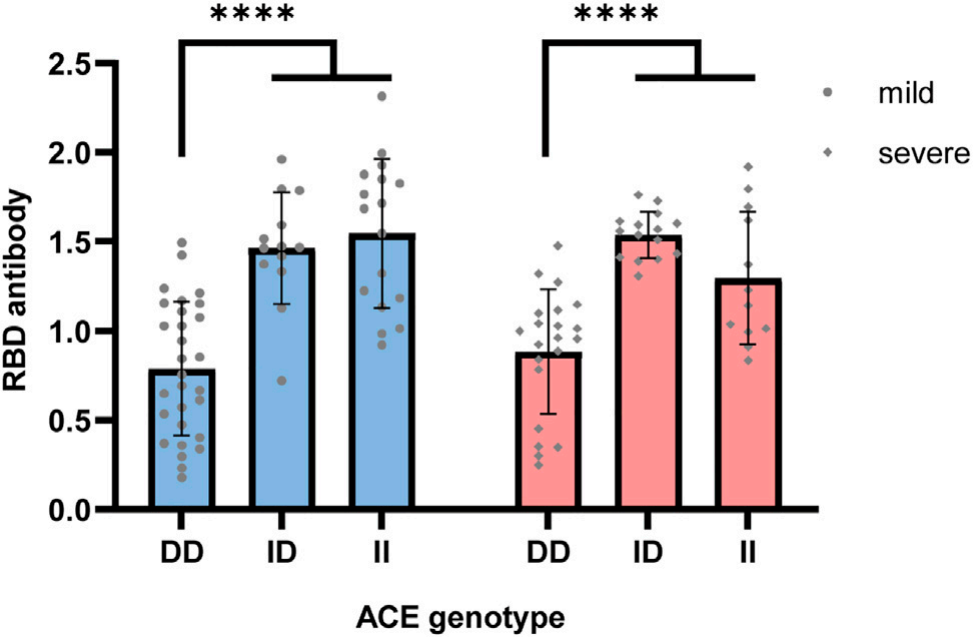
RBD antibody concentration in COVID-19 patients with different ACE genotypes. Genotype-specific RBD antibody concentration was tested with an ELISA kit, which can detect the antibody targeting on the RBD of the ACE2 receptor in the serum sample. Sample with I alleles had the highest concentration of the RBD antibody in both mild and severe disease conditions (II mild = 1.55, ID mild = 1.46; DD mild = 0.79, one-way ANOVA, *p* value < 0.0001; II severe = 1.30, ID severe = 1.54, DD severe = 0.88, one-way ANOVA, ****p value < 0.0001).

Next, we investigated the genotype-specific immune response by the concentration of the RBD antibody of SARS-COV-2 virus. We found that patients with I allele had the highest concentration of the RBD antibody in both mild and severe disease conditions [II mild = 1.55, *n* = 17; ID mild = 1.46, *n* = 13; DD mild = 0.79, *n* = 29; one-way ANOVA, F (2, 56) = 27.52, *p* value < 0.0001; II severe = 1.30, *n* = 12; ID severe = 1.54, *n* = 15; DD severe = 0.88, *n* = 21; one-way ANOVA, F (2, 45) = 20.93, *p* value < 0.0001 Figure 3]. These genotype-specific cytokine and immune responses indicated the critical role of AngII and ACE activity in RAAS during the severe symptoms caused by COVID-19 disease, which might explain the lower fatality rate and lower ICU rate of the II genotype in the Asian population.

### COVID-19 Infection Caused the Changes of Immune Pathway in Plasma Proteomics

**FIGURE 4:**
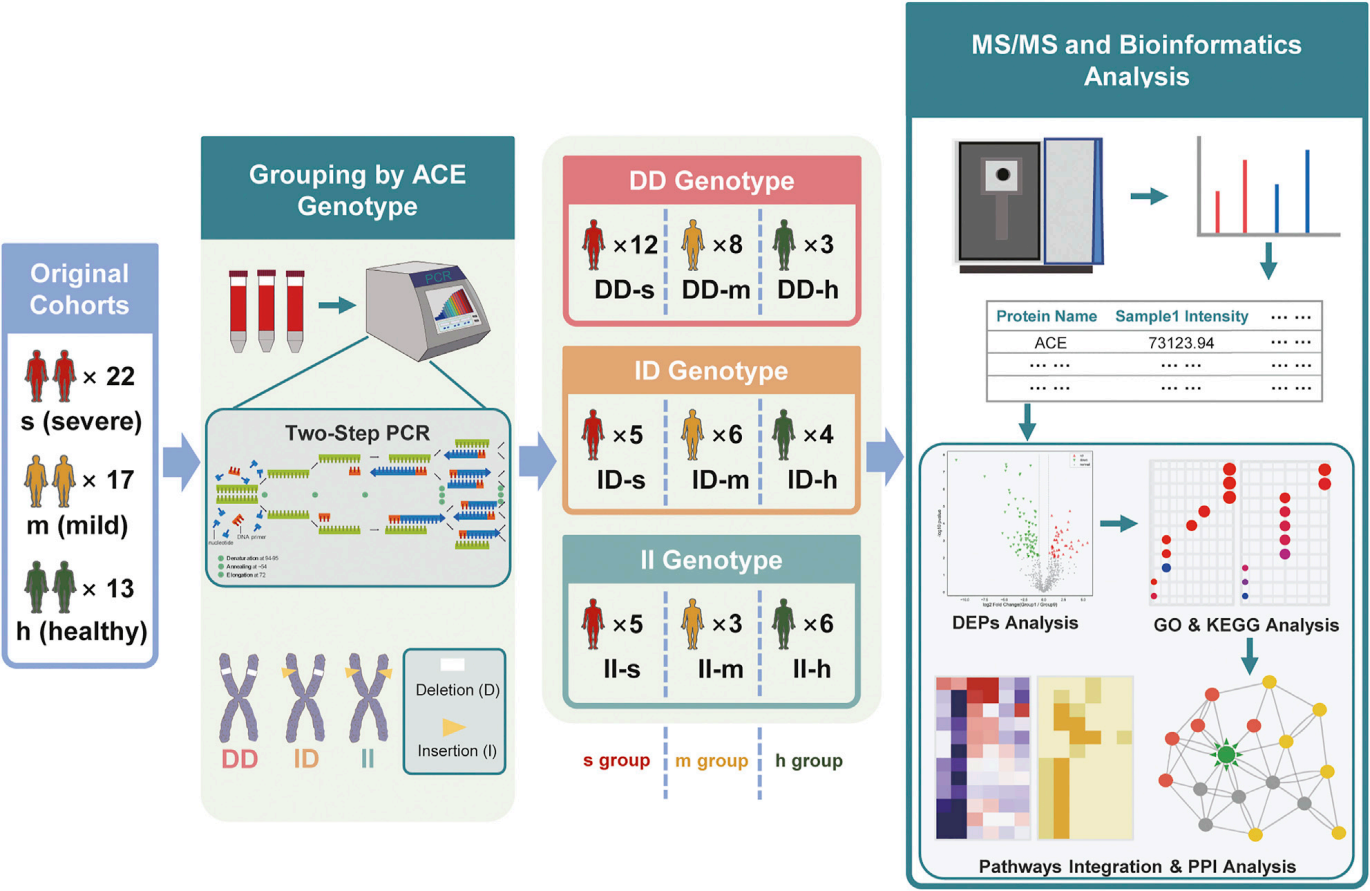
Genotyping, grouping, and proteomic analysis of prospective plasma samples. The original cohorts included 22 s (severe) samples, 17 m (mild) samples, and 13 h (healthy) samples. Each sample’s genotype was identified by the detection of the absorbance after two-step PCR. Then mass spectrometry, identification, and quantitation were adopted to analyze all the samples, followed by the bioinformatics analysis, including the DEP analysis, enrichment analysis (GO), pathway analysis (KEGG), and PPI analysis. Through multi-pathways integration and PPI analysis, biomarkers of DD genotype expression (ACE high expression) were discovered.

We collected the plasma samples of a cohort of COVID-19 patients in the prospective research, including 22 patients with severe (s) symptoms, 17 patients diagnosed with mild (m) symptoms, and 13 healthy (h) subjects at the Hubei Center for Disease Control and Prevention in Wuhan, Hubei, China. For each group, the subjects were divided into DD, ID, and II genotypes by PCR. Then we obtained 697 protein groups by performing the DIA mass spectrometry experiment. We then made 12 intra- and intergroup comparisons as follows (Figure 4).

**FIGURE 5:**
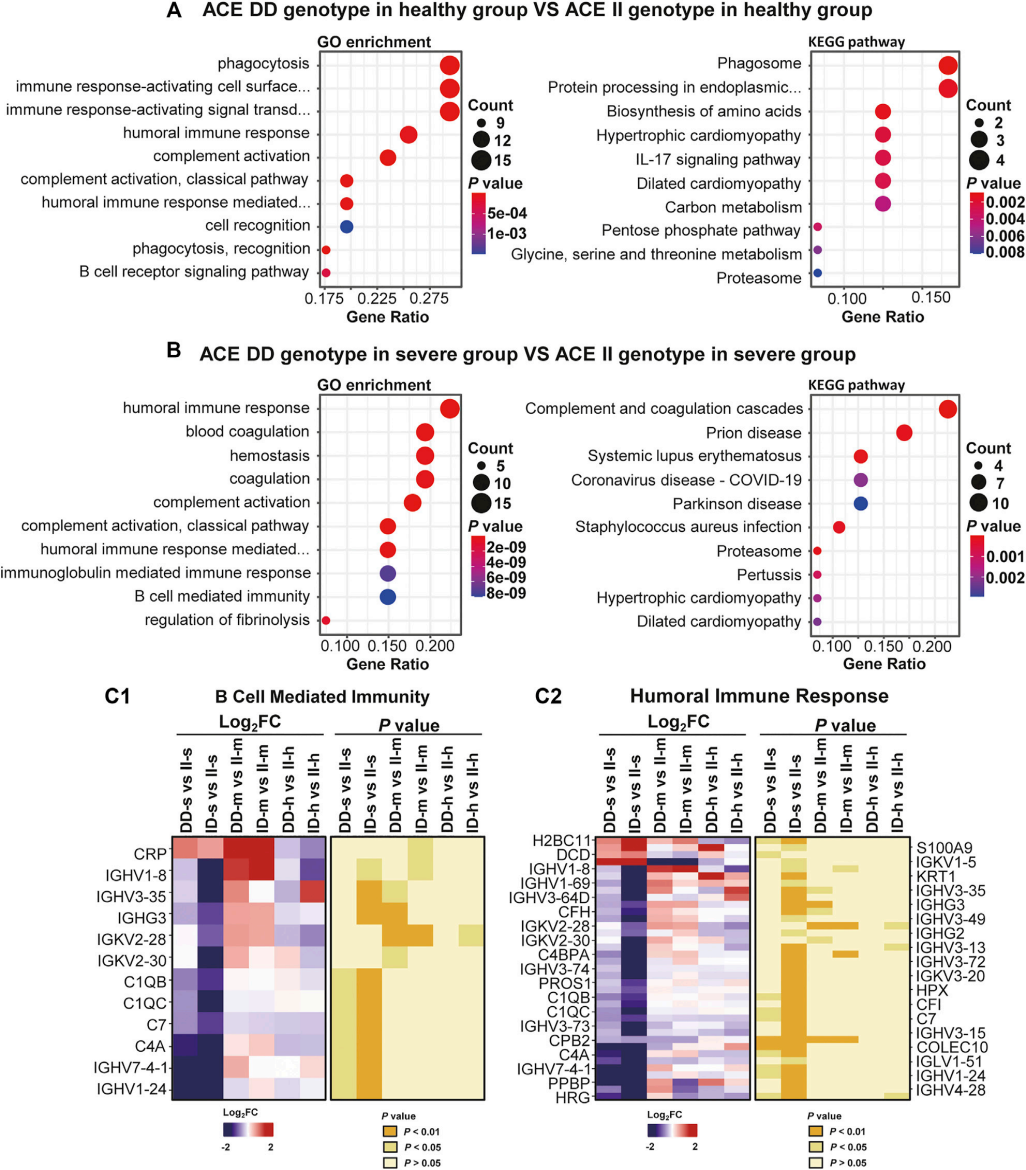
Differential expressions in severe, mild, and healthy intragroups of prospective research. **(A)** The ACE DD genotype in the healthy group vs. ACE II genotype in the healthy group. **(B)** The ACE DD genotype in severe group vs. ACE II genotype in the severe group. GO enrichment analysis (left panel) and KEGG pathway analysis (right panel) of DEPs among DD, ID, and II genotype subgroups severe and healthy intragroups. GO analysis showed the top ten biological processes (BP) sorted by *p* value. KEGG analysis shows the top 10 pathways sorted by using the *p* value. **(C1, C2)** The expressions of proteins in the B-cell–mediated immunity biological process and humoral immune response pathway in severe, mild, and healthy intragroups. Both **(C1, C2)** contained two sets of panels. The left panel shows the protein intensity ratio between different groups after log_2_ processing, with red indicating increased expression and blue indicating decreased expression. The *p* value of each ratio was calculated at the corresponding position in the right panel. Each protein in **(C1, C2)** has a fold change and a *p*-value, and just for better presentation, some are shown on the left, and the others are on the right. For **(C1)**, a total of 12 proteins are shown only on the left, and for **(C2)**, a total of 37 proteins are shown on the left and right of the figure.

First, we did intragroup comparisons and found that differences in protein expression and pathway enrichment appeared in each of them (Supplementary Table S2, Figures 5A,B). In particular, 68 and 144 differentially expressed proteins (DEPs) found in DD-s vs. II-s and ID-s vs II-s were enriched in immune-related biological processes and pathways such as B-cell–mediated immunity and humoral immune response pathways after gene ontology (GO) and Kyoto Encyclopedia of Genes and Genomes (KEGG) analyses (Figures 5C1,C2). In each pathway, immune-related proteins were enriched to show the differences in intragroup comparisons, and we found that they were significantly changed in DD and II genotypes in the severe group. Specifically, complement C1q subcomponent subunit B (C1QB), complement C1q subcomponent subunit C (C1QC), complement component C7 (C7), and complement C4-A (C4A) hardly showed any change intra-mild and healthy groups, and the similar phenomena also appeared in other immune-related pathways (Figure 5). More importantly, although both DD and II genotype patients showed many immune-related pathways (Figures 5A,B), the expression of the aforementioned proteins was significantly lower in patients with the DD genotype than in patients with the II genotype in the severe group, suggesting that patients with the II genotype produced these specific immune responses after SARS-CoV-2 invasion, which was consistent with the conclusion that the DD genotype just produced cytokine storms and II produced more immune-related responses.

**FIGURE 6:**
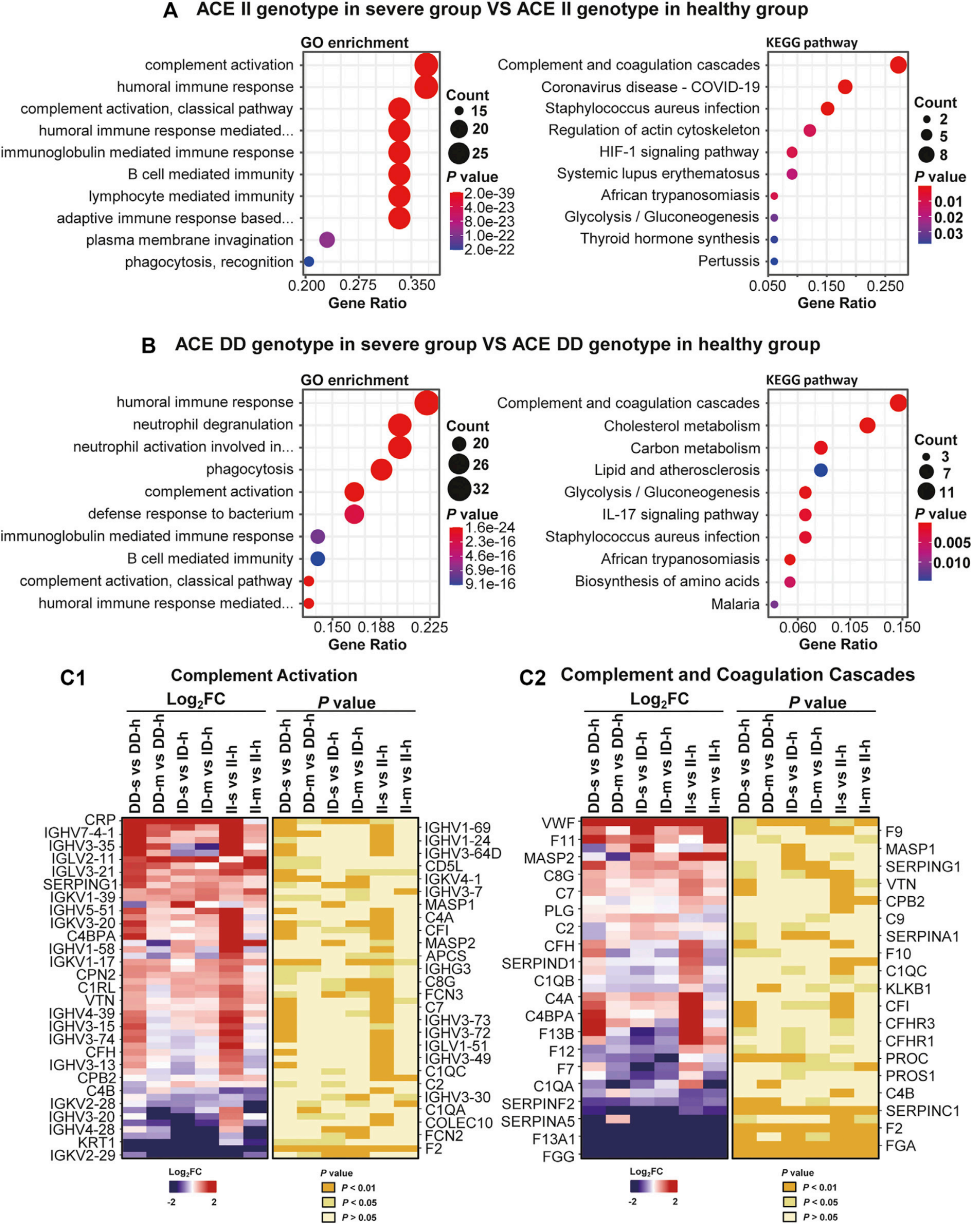
Differential expressions severe, mild, and healthy intergroups of prospective research. **(A)** The ACE II genotype in the severe group vs ACE II genotype in the healthy group. **(B)** The ACE DD genotype in the severe group vs ACE DD genotype in the healthy group. GO enrichment analysis (left panel) and KEGG pathway analysis (right panel) of DEPs severe and healthy intergroups with the same genotype. GO analysis showed the top ten biological processes sorted by using the *p* value. KEGG analysis showed the top 10 pathways sorted by *p* value. **(C1, C2)** The expressions of proteins in complement activation biological process and complement and coagulation cascades pathway in severe and mild groups divided by the healthy group. Both **(C1, C2)** contained two sets of panels. The left panel shows the protein intensity ratio between different groups after log_2_ processing, with red indicating increased expression and blue indicating decreased expression. The *p* value of each ratio was calculated at the corresponding position in the right panel. Each protein in **(C1, C2)** has a fold change and a *p* value, and just for better presentation, some are shown on the left, and the others are on the right. For **(C1, C2)**, a total of 53 and 39 proteins were shown on both the left and the right of the figures.

Second, intergroup comparisons were also performed (Supplementary Table S2, Figure 6). In these comparisons, 149 and 79 DEPs found in DD-s vs. DD-h and II-s vs. II-h were enriched in immune-related biological processes and pathways (Figures 6A,B), such as complement activation biological process and complement and coagulation cascades pathway (Figures 6C1,C2). In each pathway, immune-related proteins were displayed to show the differences in intergroup comparisons, and we found that the DEPs of samples with DD or II genotypes in comparison between the severe and healthy groups were much more significant than the comparison between the mild and healthy groups (Figure 6), and most IGHVs showed this condition, indicating that immune responses had occurred. Von Willebrand factor (VWF) was reported that it has close links to inflammation and thrombus formation (Mezger et al., 2019), and it also associated with the mortality of COVID-19 patients (Goshua et al., 2020), and upregulated significantly in all of the mild and severe groups compared with the healthy groups. Otherwise, the coagulation factors in coagulation (clotting) cascade such as F11, F9, F7, F10, and F13 were all upregulated, and they all contributed to the upregulation of coagulation. Interestingly, among the coagulation factors, F12 in the DD genotype and F2 downregulated, and for some SERPINs, dysregulation of protein expression also occurred, which showed a more significant condition in the severe group. It may cause worse coagulation disorder and other complications in the severe group. For the downregulation of F12 in the DD genotype, a similar phenomenon had occurred in previous research (D'Alessandro et al., 2020), but it did not take the ACE genotype into consideration, and for our result about the II genotype, upregulation of F11 showed the genotype specificity. Furthermore, the abnormal downregulation of F12 occurred in a pathological state and may cause compensatory upregulation of F11. Meanwhile, after the coagulation of the cascade pathway, C3 and C5 were upregulated, which indicated that the alternative pathway of complement cascade had been activated, and a non-specific defense mechanism against pathogens had been established.

To demonstrate the conclusions, retrospective research of plasma samples was performed. The plasma dataset generated from 10 batches, including six patients with fatal (F) outcomes, 12 patients diagnosed with severe (S) symptoms, 10 patients diagnosed with mild (M) symptoms, and 13 healthy (H) samples from the previous study (Shu et al., 2020), was reanalyzed (Supplementary Figure S1A). By calculating the Euclidean distance, each group was identified with the high or low ACE expression subgroup. F-l, S-l, M-l, and H-l represented the different types of subgroups with low expression of ACE. F-h, S-h, M-h, and H-h, respectively, represented different types of subgroups with high expression of ACE (Supplementary Figure S1A). Similar to the prospective research, we analyzed the DEPs intra- and inter-fatal, severe, mild, and healthy cases.

The intragroup comparison showed that many immune-related pathways of different ACE expressions were significantly changed under the influence of COVID-19 (Supplementary Figure S2B) compared with the healthy group (Supplementary Figure S2A). Furthermore, more proteins showed significant differences in the intra-fatal and severe groups than in the mild and healthy groups (Supplementary Figures S2C1,C2). Both SERPINA5 and complement factor H related 2 (CFHR2) produced significant changes in the intra-F and S groups in the humoral immune response biological progress and complement and coagulation cascades pathway, which showed no significant alteration in the M and H groups (Supplementary Figures S2C1,C2). For the intergroup analysis, the DEPs of both the inter-fatal and healthy groups with the same ACE expression enriched immune-related biological progresses and pathways (Supplementary Figures S3A,B). In further study, production of molecular mediator of immune response biological progresses (Supplementary Figure S3C1), complement and coagulation cascades, and B-cell receptor signaling pathways (Supplementary Figure S3C2) showed that in the fatal group, most proteins displayed more significant changes than the healthy group, which was advised to be influenced by the cytokine storm. All re-analyses of the data were consistent with the results in our prospective study. All of the aforementioned showed that both the expression level of ACE and COVID-19 infection were closely related to the alteration of immune-related pathways.

### COVID-19 Infection Caused the Changes in the Immune Pathway of Lung Proteomics

To further elucidate the influence of the ACE expression level on susceptibility to COVID-19, we reanalyzed the retrospective lung data (Leng et al., 2020). From these data, 5,518 protein groups were obtained. The protein intensity in the fatal (F) and the control (C) groups both showed good consistency. F and C groups were divided into two subgroups, according to the expression of ACE (Supplementary Figure S1B). F-l and C-l represent the different types of subgroups with low expression of ACE. F-h and C-h, respectively, represent different types of subgroups with high expression of ACE (Supplementary Figure S1B).

Intra- and intergroup comparisons were also carried out. In the control group, there were differences in the enrichment immune pathways under different genetic backgrounds, indicating that the susceptibility of gene polymorphism to COVID-19 was different (Supplementary Figures S4A,B); in fatal groups, more immune-related pathways were significantly enriched. However, in these pathways of the fatal group, we found that protein expression significantly changed in patients with different ACE expression categories (Supplementary Figures S4C1,C2), indicating that in the selected pathway, the immune response mechanism of different ACE expression groups was different, and the change of these pathways in the fatal group was related to the COVID-19 infection (Supplementary Figures S4A,B). Specifically, ACE with high expression in the fatal group showed significant enrichment of the neutral degeneration biological process (Supplementary Figure S4C1) in GO analysis and coronavirus disease–COVID-19 pathway in KEGG analysis (Supplementary Figure S4C2). More importantly, in the ACE high-expression group, more proteins related to COVID-19 immune pathway upregulated significantly (Supplementary Figures S4C1,C2). And for the intergroup comparisons, among the disease progresses, the fatal group showed much more significantly changed proteins and immune-related pathways than the control group (Supplementary Figure S5), which was similar to the results of retrospective plasma research. It also meant that with the aggravation of the COVID-19, the immune pathways had been enhanced.

## Discussion

In the current study, we investigated whether the ACE polymorphism is involved in the development of the COVID-19. To further explain the molecular mechanism of this polymorphism correlation, we investigated genotype-specific ACE activity, cytokine expression, and immune response from a large cohort with hundreds of COVID-19 patients.

The high serum ACE level will produce more AngII and in turn induce the high expression of ACE2, which will cause the susceptibility of SARS-CoV-2 infection (Supplementary Figure S6). Our results showed that reduced ACE activity in the COVID-19 patients may be due to the dynamic changes of ACE2 activity during the different stages of lung injury, which induce further imbalance of RAAS and accelerate the progression of the disease. In patients with mild symptoms, we found specific changes in ACE enzyme activity of gene polymorphism, which may reflect the adaptability to the disease under different gene polymorphisms, and it also explained the reason for the decrease in the severe symptom rate of gene polymorphism II. There was no difference in ACE activity among patients with severe symptoms, indicating that there was no significant difference in the regulation of ACE enzyme activity once the patients entered the severe symptomatic stage. The low-serum ACE activity in II genotype could turn out to be more useful by affecting the ACE2 function and plays a protective role in lung injury and cytokine storms.

The ACE polymorphism has been reported to be associated with cytokines such as IL-6, IL-8, and IL-10, which are biomarkers of severe COVID-19 patients (Huang et al., 2020). Characterization of the molecular basis of the susceptibility genotype in the regulation of cytokines should help explain why the risk of COVID-19 is increased in the DD patients. The DD genotype was highly correlated with COVID-19. Healthy people with the DD genotype are susceptible, and after infection, DD genotype patients have fewer antibodies and more severe cytokine storms.

To further investigate the signal pathway related to different ACE expressions in COVID-19 patients, we analyzed proteomics profiles of lung and plasma protein alternations in response to COVID-19 under different ACE expressions. Strikingly, more biomarkers are involved in the severity and death of COVID-19 patients within the higher ACE expression subgroup, suggesting that higher ACE expression is related to distinct symptoms of COVID-19. Interestingly, we also found that the COVID-19–related signal pathway was enriched in the healthy controls with higher ACE expression, indicating the DD genotype with increasing ACE activity may be the potential susceptive factor for the COVID-19. Additionally, the significantly altered proteins identified in this study are generally involved in several major biological processes, of which the identified biomarkers are included in the processes of different immune responses, platelet degranulation, and complement coagulation (Supplementary Figure S7).

It is very necessary and critical to evaluate the new drug effects on the antivirus and anti-cytokine storms of COVID-19 based on the gene polymorphisms. For example, previous studies on cancers reported that lower grade gliomas with an IDH mutation either had 1p/19q codeletion or carried a TP53 mutation. Most lower grade gliomas without an IDH mutation were molecularly and clinically similar to glioblastoma (Cancer Genome Atlas Research Network et al., 2015). In conclusion, different gene polymorphisms have different drug effects on different stages of the disease. These studies may help with designing new drugs and vaccines based on the genetic background of the population.

In conclusion, this study suggested that the ACE deletion polymorphism may be associated with the susceptibility to COVID-19 in the Chinese population. Moreover, genotype-specific immune responses and protective roles of the II genotype were found in patients infected with COVID-19. The relationship between ACE polymorphisms and COVID-19 suggests that public health institutions should further strengthen the prevention for patients with the DD genotypes, and medical institutions need to carefully assess the progression of COVID-19 patients with the DD genotypes.

## Methods and Materials

### Study Designs and Participants

This cohort study was conducted at the Hubei Center for Disease Control and Prevention in Wuhan, Hubei, China, the center of the COVID-19 outbreak. In the present study, 441 healthy and 419 COVID-19 adult Chinese patients from Hubei Province were recruited from January 2021 to March 2021 (196 male, age range, 18–80 years; 223 females: age range, 18–80 years). All of the patients were diagnosed with COVID-19 by the combination of PCR, chest CT, and antibody tests (Shu et al., 2020) (National Health Commission. 2020. Protocol on Prevention and Control of COVID-19, edition 6). To increase the ethnic homogeneity within the regions, families of patients and controls should have been residents in the region for at least two generations and all their grandparents should have been born in China. The numbers of patients with COVID-19 included in the analysis with ACE gene polymorphisms were 128 from Xiangyang, 139 from Jingmen, 71 from Xiantao, and 81 from Wuhan. All the experimental procedures including the human samples are approved by the Hubei Center for Disease Control and Prevention and Beijing Youan Hospital research ethics committee (ethical approval number: 2021-012-01).

To evaluate if the ACE polymorphism in COVID-19 patients were different from healthy controls, we used a case–control study design. For each COVID-19 patient, we matched controls by age, sex, and body mass index. Therefore, in the present study, we examined ACE polymorphism and serum concentration in 419 COVID-19 patients and 441 healthy controls. The healthy control individuals were selected among the participants from the control group of the Genetics of COVID-19 Disease China Study. The control group included individuals without personal or family history of cardiovascular diseases. Anthropometric, biochemical, clinical, and demographic characteristics, as well as cardiovascular risk factors, were determined in all controls and COVID-19 patients (hypertension: 19.3 vs. 21.2%; type 2 diabetes: 18.9 vs. 19.8%). Criteria definitions and methods have been reported previously. Obesity and hypertension definitions have already been published.

### Biosafety

All the blood samples were treated according to the biocontainment procedures of the processing of the SARS-CoV-2–positive sample.

### DNA Extraction and PCR

DNA was extracted from throat swab samples using a kit (ZTLYB-Y64, Tianlong DNA extraction kit, Wuhan, China) and stored at −80°C refrigerators in the Hubei Center of Disease Control and Prevention. The relevant fragment in intron 16 of the ACE gene was amplified according to the method of Rigat et al. (1990). Briefly, DNA was amplified in a 20 μL mixture containing 5 pmol of each primer (primer 1: 5′-TGG AGA GCC ACT CCC ATC CTT TCT-3′; primer 2: 5′-GAC GTG GCC ATC ACA TTC GTC AGA T-3′; Tianyihuiyuan, Wuhan, China), 0.5 U of Taq polymerase (TaKaRa, AJE0486A), and 2 μL of two* PCR buffers (TaKaRa) with 2.5 mM MgCl_2_ and 0.2 mM deoxyribonucleoside triphosphate. The PCR was (Eppendorf, Mastercycler X50s) ran as follows: 5-min initial denaturation at 95°C, followed by 30 cycles of 30 s at 94°C (denaturation), 30 s at 54°C (annealing), and 1 min at 72°C (extension), followed by a final elongation step at 72°C for 5 min. The presence of the D allele resulted in a 192-bp PCR product, while the I allele resulted in a 479-bp PCR product. Owing to the preferential amplification of the D allele in heterozygous samples, all samples found to have the DD genotype were included in a second insertion-specific PCR (Biller et al., 2006) (primer 1: 5′-TGG GAC CAC AGC GCC CGC CAC TAC-3′; primer 2: 5′-TCG CCA GCC CTC CCA TGC CCA TAA-3′; Tianyihuiyuan, Wuhan, China). The PCR conditions were as follows: 5-min initial denaturation at 95°C, followed by 30 cycles of 30 s at 94°C, 45 s at 62°C, and 40 s at 72°C, followed by a final elongation step at 72°C for 5 min. The presence of the I allele resulted in a 335-bp PCR product, whereas for samples homozygous for DD, no products could be detected.

### Serum ACE Measurement

Serum ACE activity was determined photometrically by commercially available kinetic tests purchased from Bühlmann Laboratories AG (Allschwil, Switzerland). Testing was performed according to the manufacturers’ instructions. ACE catalyzed the hydrolyzation of the synthetic substance N-[3- (2-furyl) acryloyl]-l-phenylalanylglycylglycine. This hydrolysis resulted in a decrease in absorbance at 340 nm, which was measured in the tests.

### Cytokine and Chemokine Measurement

To characterize the effect of coronavirus on the production of cytokines or chemokines in the acute phase of the illness based on the ACE gene polymorphism, serum cytokines and chemokines were measured using a Human Cytokine Standard 45-Plex Assay panel (R&D system, Minneapolis, United States) and the Luminex 200 system (Millipore, Boston, United States) for all patients according to the manufacturer’s instructions. The serum samples from four healthy adults were used as controls for comparisons. The median time from blood sample collection to being transferred to a designated hospital was 4 days.

### Detecting the Level of Anti–SARS-CoV-2 Spike RBD Antibodies in Serum

The principle of the kit (KIT002, Sino Biological, China) is indirect ELISA. SARS-CoV-2 (2019-nCoV) spike RBD-His recombinant protein was pre-coated onto well plate strips. The samples or control antibodies were added to the well; after incubation, the wells were washed, and a horseradish peroxidase–conjugated goat anti-human IgG was added. Following a wash, the TMB substrate solution was loaded, and colors developed in proportion to the amount of antibodies. The reaction was stopped by the addition of a stop solution, and the intensity of the color can be measured at 450 nm.

### Quantification and Statistical Analysis

Statistical analyses were performed using SPSS, version 20 (SPSS Inc., Chicago IL, United States), and Prism 8.0 (San Diego, CA, United States). Data were presented as the mean SEM. The correlation between the ACE genotype, serum ACE activity, and SARS-CoV-2 antibody was analyzed using ANOVA. Correlations were estimated using correlation matrices with Fisher’s r to z as post hoc test and Bartlett’s test of sphericity. For comparisons of the two groups, *p* values of 0.05 were regarded as statistically significant. For multiple comparisons, *p* values were corrected for the number of groups [0.5/(n-1)]. Differences between the genotype frequencies within the current study group and the expected frequencies as calculated by the Hardy–Weinberg equilibrium were estimated using chi-squared tests. Statistical analysis of the association between COVID-19 and the ACE polymorphism was calculated using logistic regression analysis (Table 1) or multiple logistic regression analysis (Table 2).

### Mass Spectrometry

Our proteomics research includes two parts: one is prospective research, including plasma proteomic analysis by using data-independent acquisition (DIA) mass spectrometry, and the other is retrospective research, including lung tissue proteomics and plasma proteomic analysis by using data-dependent acquisition (DDA) mass spectrometry.

For prospective research, the plasma samples were collected from the Xiantao Center for Disease Control and Prevention, Laohekou Center for Disease Control and Prevention, etc., and the samples were divided into three groups by the disease process, 22 for the severe group, 17 for the mild group, and 13 for the healthy group.

All fractions for DDA library generation were injected on a Thermo Scientific Q Exactive HF X mass spectrometer connected to an Easy nLC 1,200 chromatography system (Thermo Scientific). The peptide (1.5 μg) was first loaded onto an EASY-SprayTM C18 Trap column (Thermo Scientific, P/N 164946, 3 um, 75 um*2 cm), then separated on an EASY-SprayTM C18 LC Analytical Column (Thermo Scientific, ES802, 2 um, 75 um*25 cm) with a linear gradient of buffer B (80% acetonitrile and 0.1% formic acid) at a flow rate of 250 nL/min over 90 min. The MS detection method was positive, the scan range was 300–1800 m/z, resolution for MS1 scan was 60,000 at 200 m/z, the target of automatic gain control (AGC) was 3e6, maximum IT was 25 ms, and dynamic exclusion was 30.0 s. Each full MS–SIM scan followed 20 ddMS2 scans. Resolution for the MS2 scan was 15,000, AGC target was 5e4, maximum IT was 25 ms, and the normalized collision energy was 30 eV.

Each peptide sample was analyzed by LC-MS/MS operating in the data-independent acquisition (DIA) mode by Shanghai Applied Protein Technology Co., Ltd. Each DIA cycle contained one full MS–SIM scan, and 30 DIA scans covered a mass range of 350–1800 m/z with the following settings: SIM full scan resolution was 120,000 at 200 m/z; AGC, 3e6; maximum IT, 50 ms; profile mode; DIA scans were set at a resolution of 15,000; AGC target, 3e6; Max IT auto; and the normalized collision energy was 30 eV. The runtime was 90 min with a linear gradient of buffer B (80% acetonitrile and 0.1% formic acid) at a flow rate of 250 nL/min. QC samples (pooled sample from an equal aliquot of each sample in the experiment) were injected in the DIA mode at the beginning of the MS study and after every six injections throughout the experiment, which was used to monitor the MS performance.

For retrospective research, the lung data and plasma data were downloaded from the ProteomeXchange Consortium (http://proteomecentral.proteomexchange.org) via the iProX partner repository with the dataset identifier PXD018094 (http://proteomecentral.proteomexchange.org/cgi/GetDataset?ID=PXD018094) and PXD019106 (https://www.iprox.org//page/project.html?id=IPX0002173000), respectively, which was collected by Beijing Youan Hospital, Beijing, China, and Wuhan Jinyintan Hospital, Wuhan, Hubei, China. The lung tissues were collected from 11 samples, including three COVID-19-infected lung samples from the fatal group and eight paracancerous tissues of lung cancer without COVID-19 pneumonia samples from the control group. A total of 10 batches of TMT 11 plex labeling experiments (both cohort 1 and cohort 2) were involved in the plasma dataset.

### Proteomics Data Analysis

#### Prospective Analysis

DDA data were imported directly into Spectronaut software (SpectronautTM 14.4.200727.47784) to structure the spectral library. The database was downloaded from the website http://www.uniprot.org. iRT sequence was added. The parameters were set as follows: enzyme was trypsin, max missed cleavage was two, fixed modification was carbamidomethyl (C), and dynamic modification was oxidation (M) and acetyl (protein N-term). All reported data were based on 99% confidence for protein identification as determined by the false discovery rate {FDR = N (decoy)*2/[N (decoy)+ N (target)]} ≤ 1%. The spectral library was constructed by importing the original raw files and DDA searching results into Spectronaut Pulsar X TM_12.0.20491.4 (Biognosys).

DIA data were analyzed with Spectronaut Pulsar XTM searching the aforementioned constructed spectral library. Main software parameters were set as follows: retention time prediction type was dynamic iRT, interference on MS2 level correction was enabled, and cross-run normalization was enabled. All results were filtered based on a q-value cutoff of 0.01 (equivalent to FDR<1%).

#### Retrospective Analysis

All raw data were analyzed by pFind (Chi et al., 2018) and pQuant with parameters similar to the mentioned articles. The raw data were searched against the human proteome database containing the Swiss-Prot sequences (downloaded from the UniProt database on March 21, 2020, containing 75,116 proteins for lung tissue dataset and on August 17, 2020, containing 20,387 proteins for plasma dataset). For lung tissues and plasma datasets, open-search mod was activated in pFind to achieve high MS/MS interpretation rate. We used pQuant label-free workflow to analyze the lung tissue dataset and TMT workflow for the plasma dataset.

### Proteomics Statistical Analysis

For the quantified proteins, the median normalization method was used to reduce the biases between samples. To obtain the significantly changed proteins, named differentially expressed proteins (DEPs) in this article, the *p* value was calculated for all samples, using unpaired two-sided Welch’s *t*-test, and the fold change was calculated based on the ratio of two groups. The results were filtered by using the *p* value <0.05 and fold change >1.5 or <1.5^–1^. The UniProtKB/Swiss-Prot public database was used to map the gene names of DEPs, and clusterProfiler (v.3.18.0) in R studio (v.February 1, 1.2.5033) was used to enrich GO terms and KEGG pathways. STRING (v.11.0) and Cytoscape (v.3.7.2) were used to generate the protein–protein interaction (PPI) plot.

## Data Availability

The original contributions presented in the study are publicly available in the ProteomeXchange Consortium (http://proteomecentral.proteomexchange.org) *via* the iProX partner repository with the dataset identifier PXD028015.

## References

[B1] Bhatraju P. K. Ghassemieh B. J. Nichols M. Kim R. Jerome K. R. Nalla A. K. (2020). Covid-19 in Critically Ill Patients in the Seattle Region - Case Series. N. Engl. J. Med. 382, 2012–2022. 10.1056/NEJMoa2004500 32227758 PMC7143164

[B2] Biller H. Zissel G. Ruprecht B. Nauck M. Busse Grawitz A. Müller-Quernheim J. (2006). Genotype-Corrected Reference Values for Serum Angiotensin-Converting Enzyme. Eur. Respir. J. 28, 1085–1090. 10.1183/09031936.00050106 17138677

[B3] Boright A. P. Paterson A. D. Mirea L. Bull S. B. Mowjoodi A. Scherer S. W. (2005). Genetic Variation at the ACE Gene Is Associated with Persistent Microalbuminuria and Severe Nephropathy in Type 1 Diabetes: the DCCT/EDIC Genetics Study. Diabetes 54, 1238–1244. 10.2337/diabetes.54.4.1238 15793268 PMC1621110

[B4] Cambien F. Poirier O. Lecerf L. Evans A. Cambou J. P. Arveiler D. (1992). Deletion Polymorphism in the Gene for Angiotensin-Converting Enzyme Is a Potent Risk Factor for Myocardial Infarction. Nature 359, 641–644. 10.1038/359641a0 1328889

[B5] Cancer Genome Atlas Research Network Brat D. J. Verhaak R. G. Aldape K. D. Yung W. K. Salama S. R. (2015). Comprehensive, Integrative Genomic Analysis of Diffuse Lower-Grade Gliomas. N. Engl. J. Med. 372, 2481–2498. 10.1056/NEJMoa1402121 26061751 PMC4530011

[B6] Chan J. F. Yuan S. Kok K. H. To K. K. Chu H. Yang J. (2020). A Familial Cluster of Pneumonia Associated with the 2019 Novel Coronavirus Indicating Person-To-Person Transmission: a Study of a Family Cluster. Lancet 395, 514–523. 10.1016/S0140-6736(20)30154-9 31986261 PMC7159286

[B7] Chi H. Liu C. Yang H. Zeng W-F. Wu L. Zhou W-J. (2018). Comprehensive Identification of Peptides in Tandem Mass Spectra Using an Efficient Open Search Engine. Nat. Biotechnol. 36, 1059–1061. 10.1038/nbt.4236 30295672

[B8] D'Alessandro A. Thomas T. Dzieciatkowska M. Hill R. C. Francis R. O. Hudson K. E. (2020). Serum Proteomics in COVID-19 Patients: Altered Coagulation and Complement Status as a Function of IL-6 Level. J. Proteome Res. 19, 4417–4427. 10.1021/acs.jproteome.0c00365 32786691 PMC7640953

[B9] Ghafouri-Fard S. Noroozi R. Vafaee R. Branicki W. Poṡpiech E. Pyrc K. (2020). Effects of Host Genetic Variations on Response to, Susceptibility and Severity of Respiratory Infections. Biomed. Pharmacother. 128, 110296. 10.1016/j.biopha.2020.110296 32480226 PMC7258806

[B10] Goshua G. Pine A. B. Meizlish M. L. Chang C. H. Zhang H. Bahel P. (2020). Endotheliopathy in COVID-19-Associated Coagulopathy: Evidence from a single-centre, Cross-Sectional Study. Lancet Haematol. 7, e575–e582. 10.1016/S2352-3026(20)30216-7 32619411 PMC7326446

[B11] Hirano T. Murakami M. (2020). COVID-19: A New Virus, but a Familiar Receptor and Cytokine Release Syndrome. Immunity 52, 731–733. 10.1016/j.immuni.2020.04.003 32325025 PMC7175868

[B12] Huang C. Wang Y. Li X. Ren L. Zhao J. Hu Y. (2020). Clinical Features of Patients Infected with 2019 Novel Coronavirus in Wuhan, China. Lancet 395, 497–506. 10.1016/S0140-6736(20)30183-5 31986264 PMC7159299

[B13] Huang R. F. Dong P. Zhang T. Z. Ying X. J. Hu H. (2016). Angiotensin-converting Enzyme Insertion/Deletion Polymorphism and Susceptibility to Allergic Rhinitis in Chinese Populations: A Systematic Review and Meta-Analysis. Eur. Arch. Otorhinolaryngol. 273, 277–283. 10.1007/s00405-014-3350-6 25341696

[B14] John R. Dhillon M. S. Dhillon S. (2020). Genetics and the Elite Athlete: Our Understanding in 2020. Indian J. Orthop. 54, 256–263. 10.1007/s43465-020-00056-z 32399143 PMC7205921

[B15] Kuba K. Imai Y. Rao S. Gao H. Guo F. Guan B. (2005). A Crucial Role of Angiotensin Converting Enzyme 2 (ACE2) in SARS Coronavirus-Induced Lung Injury. Nat. Med. 11, 875–879. 10.1038/nm1267 16007097 PMC7095783

[B16] Leng L. Cao R. Ma J. Mou D. Zhu Y. Li W. (2020). Pathological Features of COVID-19-Associated Lung Injury: a Preliminary Proteomics Report Based on Clinical Samples. Signal. Transduct Target. Ther. 5, 240. 10.1038/s41392-020-00355-9 33060566 PMC7557250

[B17] Mahmudpour M. Roozbeh J. Keshavarz M. Farrokhi S. Nabipour I. (2020). COVID-19 Cytokine Storm: The Anger of Inflammation. Cytokine 133, 155151. 10.1016/j.cyto.2020.155151 32544563 PMC7260598

[B18] Mezger M. Nording H. Sauter R. Graf T. Heim C. von Bubnoff N. (2019). Platelets and Immune Responses During Thromboinflammation. Front. Immunol. 10, 1731. 10.3389/fimmu.2019.01731 31402914 PMC6676797

[B19] Pachocka L. Włodarczyk M. Kłosiewicz-Latoszek L. Stolarska I. (2020). The Association between the Insertion/deletion Polymorphism of the Angiotensin Converting Enzyme Gene and Hypertension, as Well as Environmental, Biochemical and Anthropometric Factors. Rocz Panstw Zakl Hig 71, 207–214. 10.32394/rpzh.2020.0119 32519825

[B20] Pacurari M. Kafoury R. Tchounwou P. B. Ndebele K. (2014). The Renin-Angiotensin-Aldosterone System in Vascular Inflammation and Remodeling. Int. J. Inflam 2014, 689360. 10.1155/2014/689360 24804145 PMC3997861

[B21] Raba G. Zawlik I. Braun M. Paszek S. Potocka N. Skrzypa M. (2020). Evaluation of the Association between Angiotensin Converting Enzyme Insertion/deletion Polymorphism and the Risk of Endometrial Cancer in and Characteristics of Polish Women. Adv. Clin. Exp. Med. 29, 581–585. 10.17219/acem/118843 32442362

[B22] Rigat B. Hubert C. Alhenc-Gelas F. Cambien F. Corvol P. Soubrier F. (1990). An Insertion/Deletion Polymorphism in the Angiotensin I-Converting Enzyme Gene Accounting for Half the Variance of Serum Enzyme Levels. J. Clin. Invest. 86, 1343–1346. 10.1172/JCI114844 1976655 PMC296868

[B23] Sayed-Tabatabaei F. A. Houwing-Duistermaat J. J. van Duijn C. M. Witteman J. C. (2003). Angiotensin-Converting Enzyme Gene Polymorphism and Carotid Artery Wall Thickness: a Meta-Analysis. Stroke 34, 1634–1639. 10.1161/01.STR.0000077926.49330.64 12805498

[B24] Sharma P. (1998). Meta-Analysis of the ACE Gene in Ischaemic Stroke. J. Neurol. Neurosurg. Psychiatry 64, 227–230. 10.1136/jnnp.64.2.227 9489536 PMC2169971

[B25] Shu T. Ning W. Wu D. Xu J. Han Q. Huang M. (2020). Plasma Proteomics Identify Biomarkers and Pathogenesis of COVID-19. Immunity 53, 1108–e5. 10.1016/j.immuni.2020.10.008 33128875 PMC7574896

[B26] Yang G. Tan Z. Zhou L. Yang M. Peng L. Liu J. (2020). Effects of Angiotensin II Receptor Blockers and ACE (Angiotensin-Converting Enzyme) Inhibitors on Virus Infection, Inflammatory Status, and Clinical Outcomes in Patients with COVID-19 and Hypertension: A Single-Center Retrospective Study. Hypertension 76, 51–58. 10.1161/HYPERTENSIONAHA.120.15143 32348166

[B27] Zhu N. Zhang D. Wang W. Li X. Yang B. Song J. (2020). A Novel Coronavirus from Patients with Pneumonia in China, 2019. N. Engl. J. Med. 382, 727–733. 10.1056/NEJMoa2001017 31978945 PMC7092803

