# Supplementary material for: Angiotensin-Converting Enzyme Genotype–Specific Immune Response Contributes to the Susceptibility of COVID-19: A Nested Case–Control Study
Source: Front Pharmacol. 2022 Jan 12;12:759587. doi: 10.3389/fphar.2021.759587 (PMC8790029; doi:10.3389/fphar.2021.759587)
Supplement: Supplementary file 1 [file DataSheet1.pdf]

Angiotensin-converting enzyme genotype specific immune response contributes to the susceptibility of COVID-19

Pengyun Gong [1†], Fanghua Mei [2†], Ruili Li [3†], Yuchen Wang [1], Weizheng Li [1], Kai Pan [2], Junqiang Xu [2], Chao Liu1 [*], Hongjun Li [3*], Kun Cai [2*], Wei Shi [1*]

[1] School of Engineering Medicine & School of Biological Science and Medical Engineering, Beihang University, Beijing 100191, China

[2] Hubei Center for Disease Control and Prevention, Wuhan 430079, China.

[3] Department of Radiology, Beijing YouAn Hospital, Capital Medical University, Beijing 100069, China

[†] These authors contributed equally to this work.

[*] Correspondence should be addressed to Wei Shi (shiweilab@buaa.edu.cn) or Kun Cai (ckreal@163.com) or Hongjun Li (lihongjun00113@ccmu.edu.cn) or Chao Liu (liuchaobuaa@buaa.edu.cn).

Table of Contents

Table S1 Demographic, clinical parameters in COVID-19 patients and matched controls. 1

Table S2 Distribution of ACE genotype in the different symptom of COVID-19 patients according to risk status. 2

Figure S1 Retrospective Plasma samples grouped by ACE. 3

Figure S2 Differential expressions intra fatal, severe, mild and healthy groups of retrospective plasma samples. 4

Figure S3 Differential expressions inter fatal, severe, mild and healthy groups of retrospective research. 6

Figure S4 Differential expressions intra fatal and healthy groups of retrospective lung samples. 8

Figure S5 Differential expressions inter fatal and control groups of retrospective research. 10

Fig.S6 A possible mechanism for the ACE dependent susceptibility to COVID-19.......... 12

Figure S7 Protein-Protein Interaction Network of Enriched Immune Responses 13

Key resource tables 15

Table S1 Demographic, clinical parameters in COVID-19 patients and matched controls.

	COVID-19 (n=419)	Healthy control (n=441)	P value
**Male (%)**	46.8	49.6	0.51
**Age (years)**	50.2±16.1	49.1±8.7	0.51
**BMI (kg/m^2)**	23.08	24.02	0.52
**Hypertension (%)**	21.2	19.3	0.49
**Type 2 diabetes (%)**	19.8	18.9	0.50

Table S2 Distribution of ACE genotype in the different symptom of COVID-19 patients according to risk status.

Prospective Plasma Samples				Retrospective Plasma Samples				Retrospective Lung Tissue Samples			
Number of identified protein groups: 697				Number of identified protein groups: 767				Number of identified protein groups: 5518			
Number of intra groups		Number of inter groups		Number of intra groups		Number of inter groups		Number of intra groups		Number of inter groups	
DD-s vs II-s	68	DD-s vs DD-h	149	F-h vs F-l	98	F-h vs H-h	365	F-h vs F-l	131	F-h vs C-h	1619
ID-s vs II-s	144	DD-m vs DD-h	231	S-h vs S-l	70	S-h vs H-h	334	C-h vs C-l	530	F-l vs C-l	2557
DD-m vs II-m	55	ID-s vs ID-h	212	M-h vs M-l	16	M-h vs H-h	179				
ID-m vs II-m	38	ID-m vs ID-h	216	H-h vs H-l	75	F-l vs H-l	335				
DD-h vs II-h	53	II-s vs II-h	79			S-l vs H-l	221				
ID-h vs II-h	41	II-m vs II-h	188			M-l vs H-l	208				

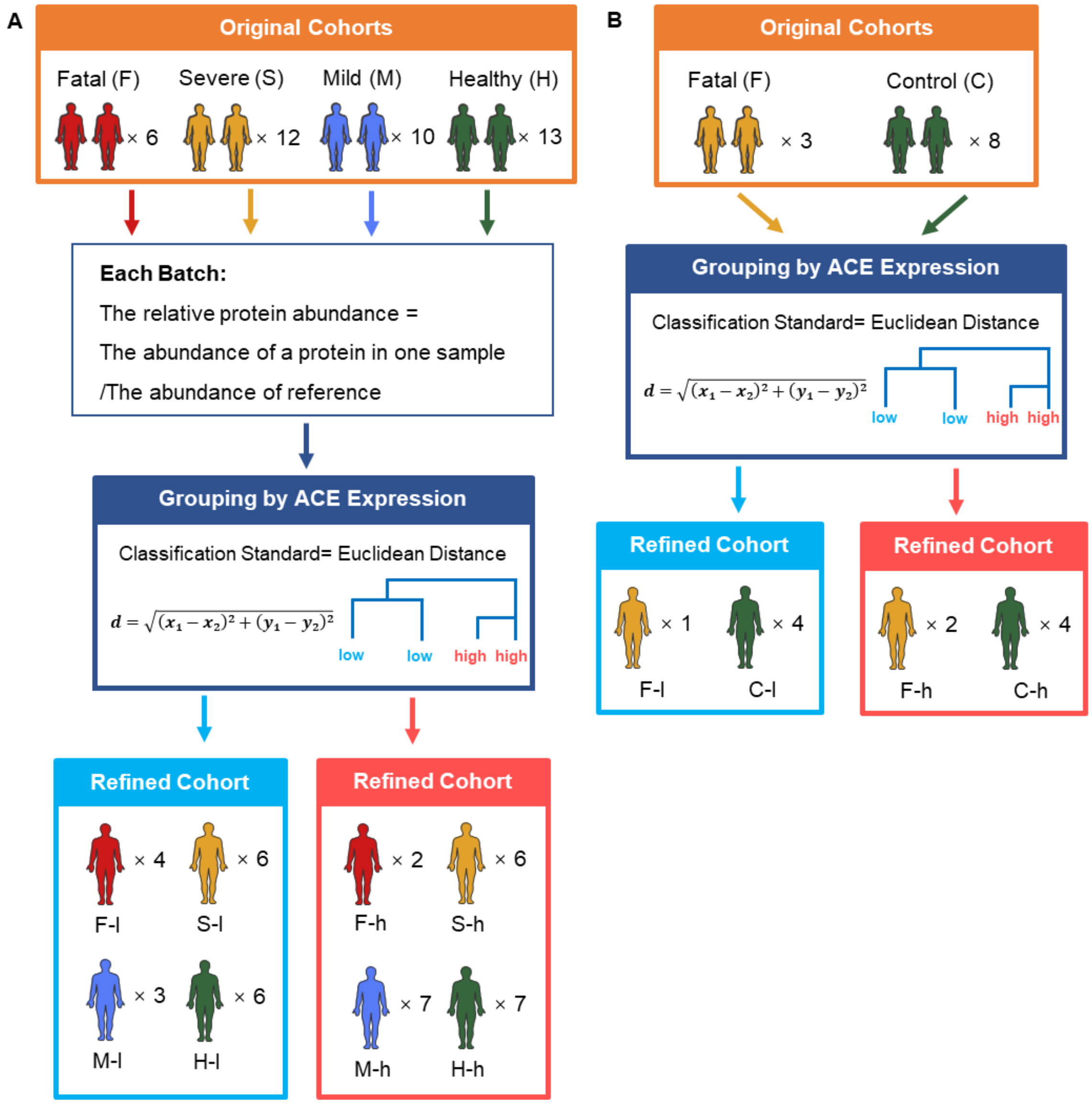

Figure S1 Retrospective Plasma samples grouped by ACE.

According to the Euclidean distance between ACE gene expression, the original groups were divided into high ACE expression sub-groups and low ACE expression sub-groups.

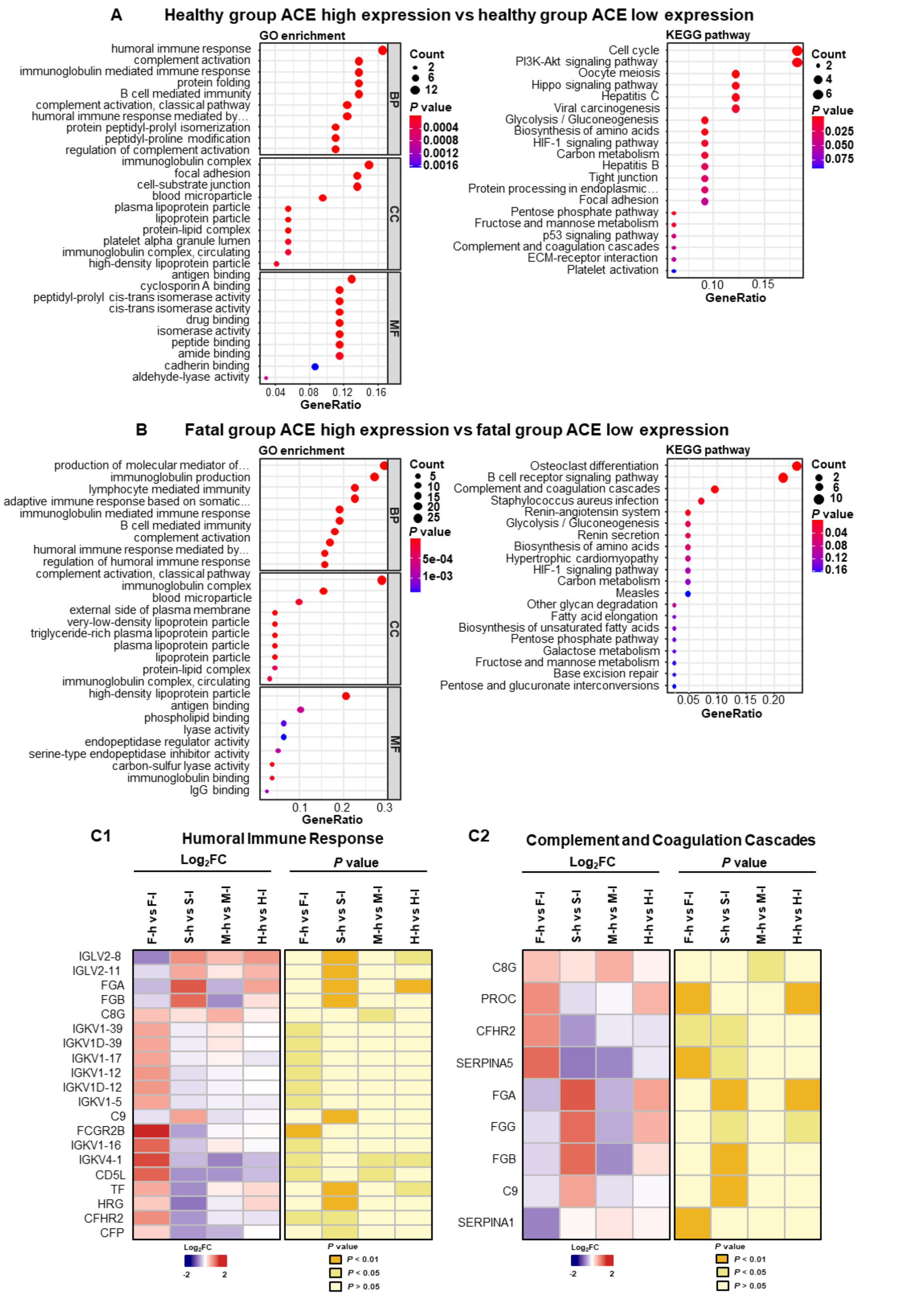

Figure S2 Differential expressions intra fatal, severe, mild and healthy groups of retrospective plasma samples.

(A) Healthy group ACE high expression vs healthy group ACE low expression. (B)

Fatal group ACE high expression vs fatal group ACE low expression. GO enrichment analysis (left panel) and KEGG pathway analysis (right panel) of DEPs between high and low ACE expression sub-groups intra fatal and healthy groups. GO analysis showed the top ten terms sorted by *P* value in biological progress (BP), cellular component (CC) and molecular function (MF), KEGG analysis showed the top 20 pathways sorted by *P* value. (C1) (C2) The expressions of proteins in Humoral Immune Response biological process and Complement and Coagulation Cascades pathway intra fatal, severe, mild and healthy group.

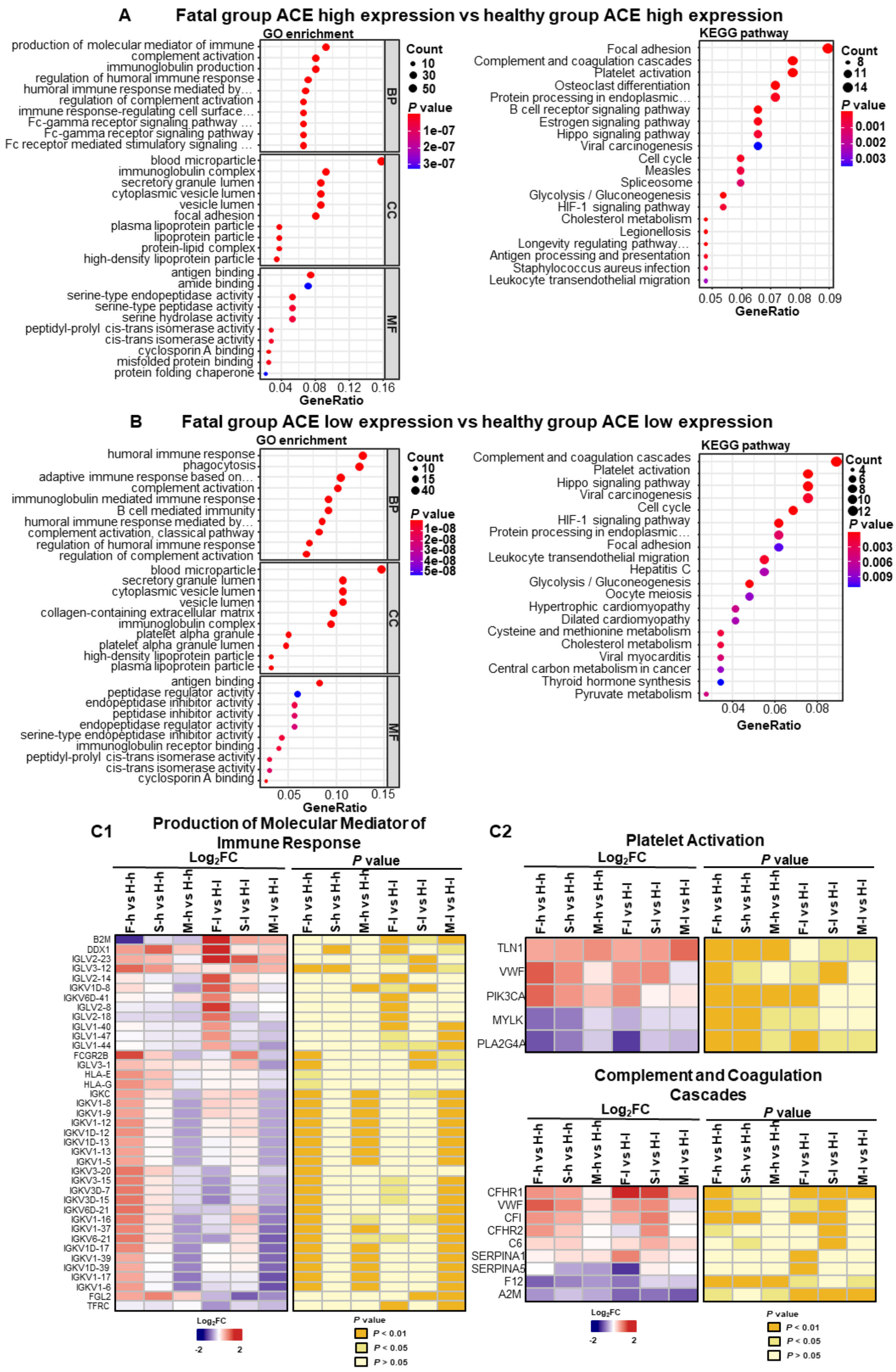

Figure S3 Differential expressions inter fatal, severe, mild and healthy groups of retrospective research.

(A) Fatal group ACE high expression vs healthy group ACE high expression. (B)

Fatal group ACE low expression vs healthy group ACE low expression. GO enrichment analysis (left panel) and KEGG pathway analysis (right panel) of DEPs inter fatal and healthy groups with the same ACE expression level. GO analysis showed the top ten terms sorted by *P* value in biological progress (BP), cellular component (CC) and molecular function (MF), KEGG analysis showed the top 20 pathways sorted by *P* value. (C1) (C2) The expressions of proteins in Production of Molecular Mediator of Immune Response biological process and B Cell Receptor Signaling pathway in fatal, severe and mild groups divided by healthy group.

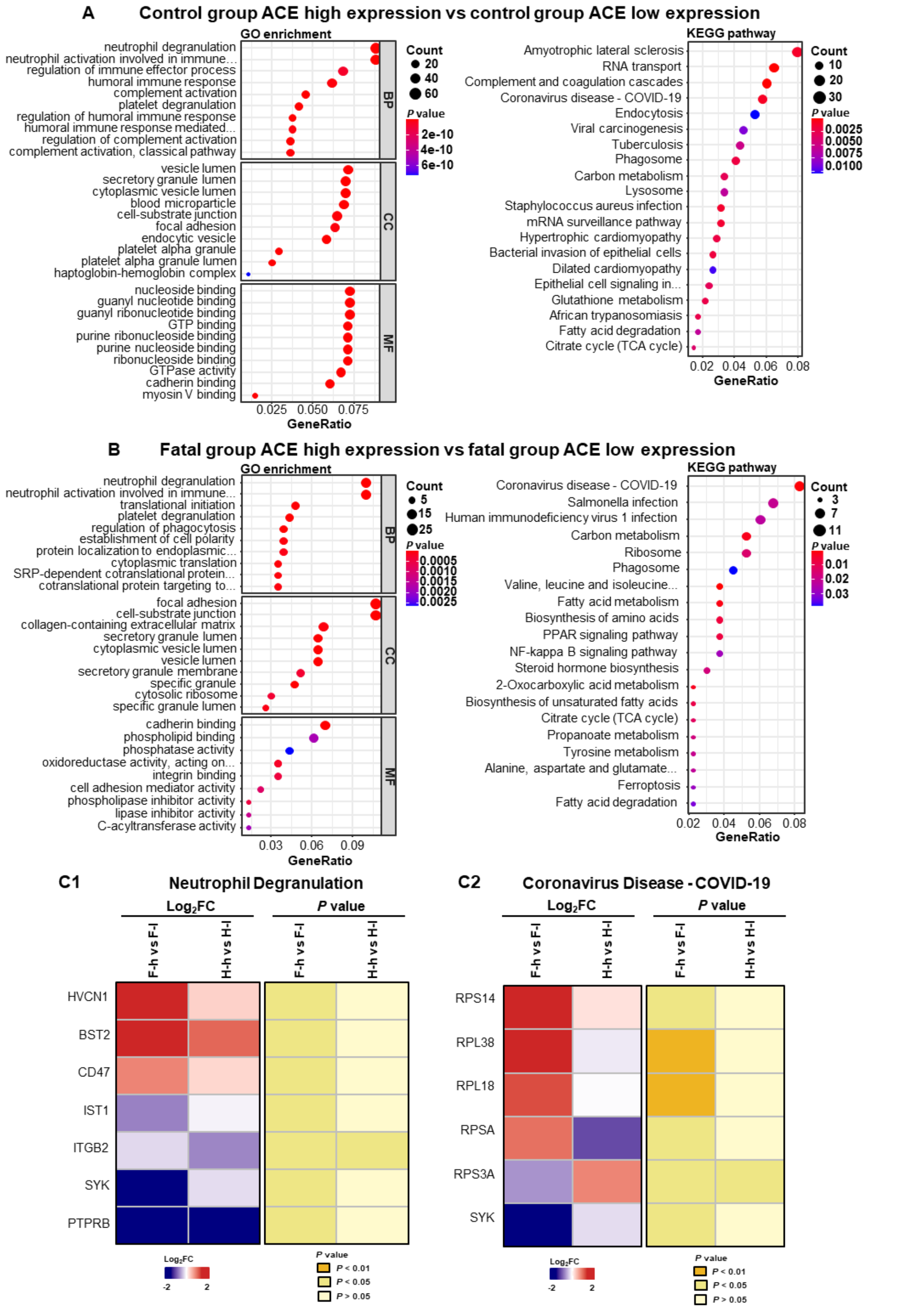

Figure S4 Differential expressions intra fatal and healthy groups of retrospective lung samples.

(A) Control group ACE high expression vs control group ACE low expression. (B)

Fatal group ACE high expression vs fatal group ACE low expression. GO enrichment analysis (left panel) and KEGG pathway analysis (right panel) of DEPs between high and low ACE expression sub-groups intra fatal and control groups. GO analysis showed the top ten terms sorted by *P* value in biological progress (BP), cellular component (CC) and molecular function (MF), KEGG analysis showed the top 20 pathways sorted by *P* value. (C1) (C2) The expressions of proteins in Neutrophil Degranulation biological process and Coronavirus Disease-COVID-19 pathway intra fatal and control group.

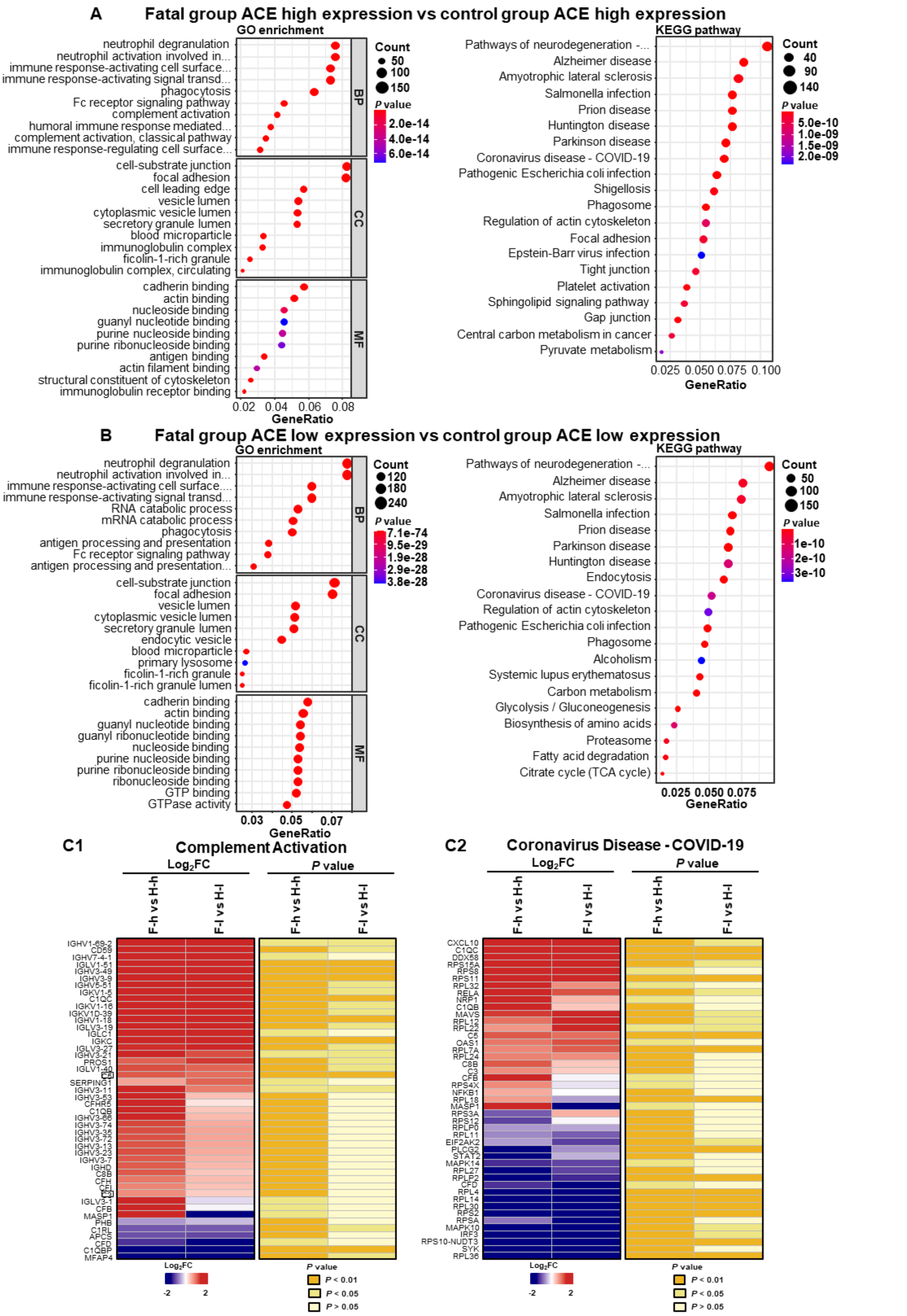

Figure S5 Differential expressions inter fatal and control groups of retrospective research.

(A) Fatal group ACE high expression vs control group ACE high expression. (B)

Fatal group ACE low expression vs control group ACE low expression. GO enrichment analysis (left panel) and KEGG pathway analysis (right panel) of DEPs inter fatal and control groups with the same ACE expression level. GO analysis showed the top ten terms sorted by *P* value in biological progress (BP), cellular component (CC) and molecular function (MF), KEGG analysis showed the top 20 pathways sorted by *P* value. (C1) (C2) The expressions of proteins in Complement Activation biological process and Coronavirus Disease-COVID-19 pathway in fatal group divided by control group.

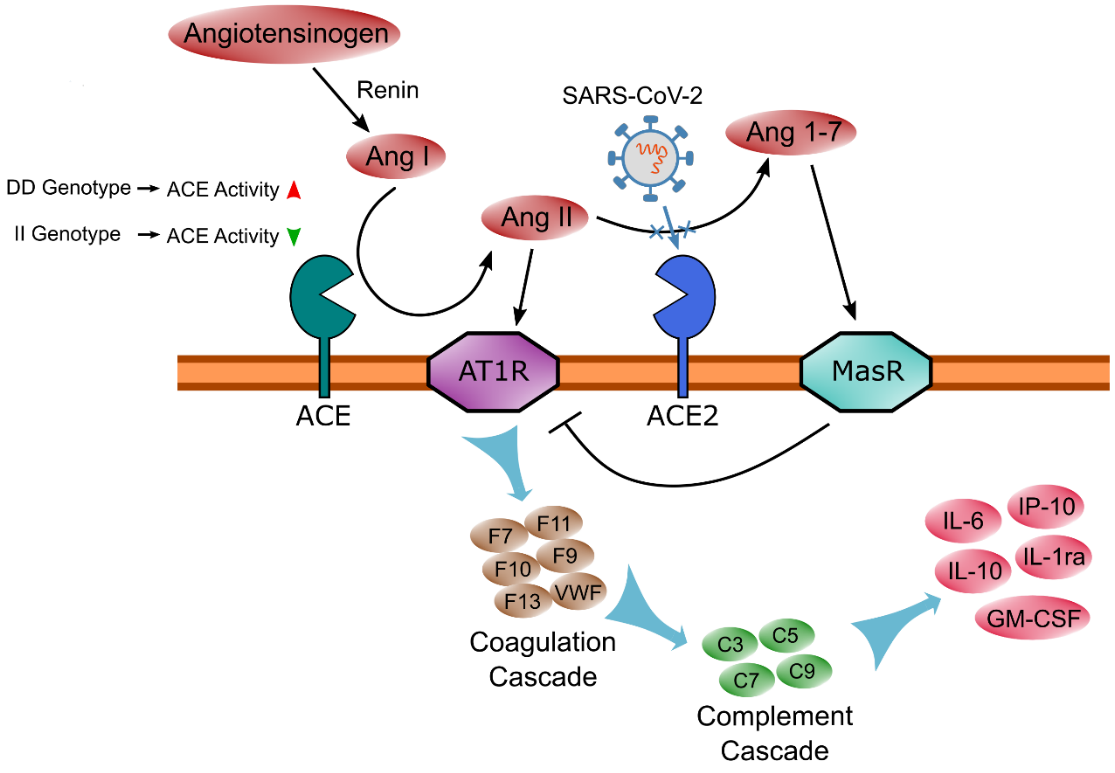

Fig.S6 A possible mechanism for the ACE dependent susceptibility to COVID-19

Once ACE2 was occupied by the virus infection, AngII would be accumulated and activate the immune response mediated by AT1R. The Coagulation Cascade involving F7, F11, F9, VWF, F10 and F13 will be activated, the up-regulation of C3, C5, C7 and C9 would lead to the activation of Alternative pathway of Complement Cascade, and then inflammatory factors will be produced. As ACE2 did not convert AngII into Ang1-7, it would be difficult to activate MasR to control inflammation. Otherwise, the patients with ACE DD genotype had the higher ACE activity, and the patients with ACE II genotype showed the lower ACE activity.

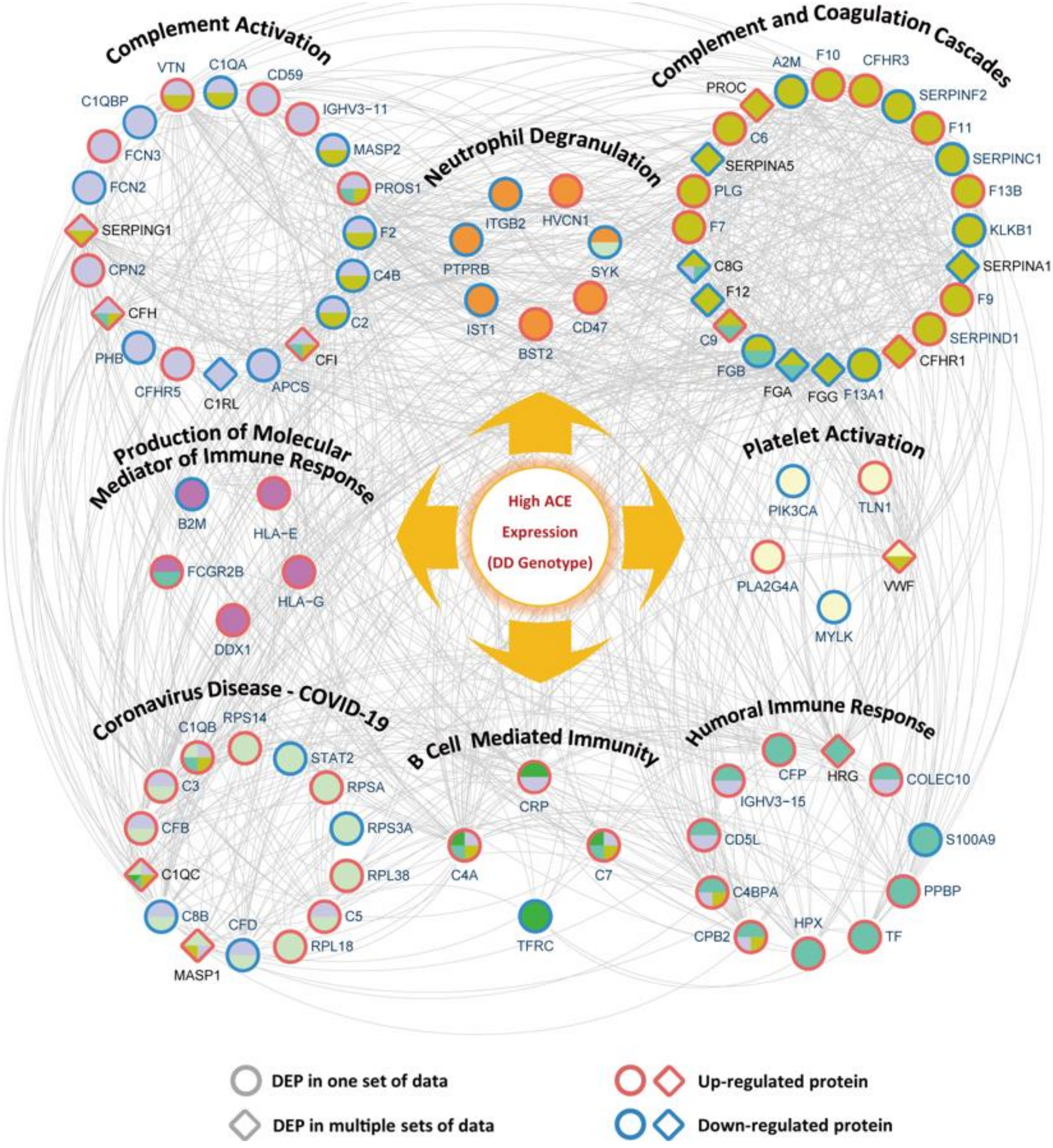

Figure S7 Protein-Protein Interaction Network of Enriched Immune Responses

149 DEPs in retrospective research and 94 DEPs in prospective research were enriched into 8 immune response pathways on the basis of their major functions when DD genotype ACE protein was highly expressed, including Complement Activation, Neutrophil Degranulation, Complement and Coagulation Cascades, Production of Molecular Mediator of Immune Response, Platelet Activation, Coronavirus Disease-Covid-19, B Cell Mediated Immunity and Humoral Immune Response. For each pathway, the most significant proteins are showed in the network. Each immune response was represented by a solid circle or diamond with different colors. Red edge

represented the up-regulated protein, and blue edge represented the down-regulated one.

Key resource tables

Reagent or resource	source	identifier
Blood samples from human patients infected with SARS-CoV-2	This paper	N/A
Blood samples from healthy donors	This paper	N/A
**Commercial assays**		
DNA extraction kit	Tianlong DNA extraction kit	ZTLYB-Y64
serum ACE activity	Bühlmann Laboratories AG	KK-ACK
Human Cytokine Standard 45-Plex Assays panel	R&D system	LKTM014
RBD antibody-detection assay ELISA kit	Sino biological	KIT002
**Data and software**		
lung tissues proteomics data	http://proteomecentral.proteomexchange.org/cgi/GetDataset?ID=PXD018094	PXD018094
plasma proteomics data	https://www.iprox.org//page/project.html?id=IPX0002173000	PXD019106
pFind and pQuant	http://pfind.ict.ac.cn	version 3.0
